# Transgenic Mice Expressing Porcine Prion Protein Resistant to Classical Scrapie but Susceptible to Sheep Bovine Spongiform Encephalopathy and Atypical Scrapie

**DOI:** 10.3201/eid1508.081218

**Published:** 2009-08

**Authors:** Juan-Carlos Espinosa, María-Eugenia Herva, Olivier Andréoletti, Danielle Padilla, Caroline Lacroux, Hervé Cassard, Isabelle Lantier, Joaquin Castilla, Juan-María Torres

**Affiliations:** Centro de Investigación en Sanidad Animal, Madrid, Spain (J.-C. Espinosa, M.-E. Herva, D. Padilla, J. Castilla, J.-M. Torres); École Nationale Vétérinaire de Toulouse, Toulouse, France (O. Andréoletti, C. Lacroux, H. Cassard); Centre Institut National de la Recherche Agronomique de Tours, Nouzilly, France (I. Lantier); 1 These authors contributed equally to this article.

**Keywords:** Porcine prion, bovine spongiform encephalopathy, sheep, scrapie, transmission barrier, prions and related diseases, atypical scrapie, research

## Abstract

How susceptible pigs are to infection with sheep prions is unknown. We show, through transmission experiments in transgenic mice expressing porcine prion protein (PrP), that the susceptibility of this mouse model to bovine spongiform encephalopathy (BSE) can be enhanced after its passage in ARQ sheep, indicating that the pathogenicity of the BSE agent is modified after passage in sheep. Transgenic mice expressing porcine PrP were, nevertheless, completely resistant to infection with a broad panel of classical scrapie isolates from different sheep PrP genotypes and with different biochemical characteristics. The atypical (Nor98 like) isolate (SC-PS152) was the only scrapie isolate capable of transmission in these mice, although with a marked transmission barrier. Unexpectedly, the atypical scrapie agent appeared to undergo a strain phenotype shift upon transmission to porcine-PrP transgenic mice and acquired new strain properties, suggesting that atypical scrapie agent may exhibit different phenotypes depending on the host cellular PrP or other genetic factors.

## Abstract 2

Atypical scrapie strain phenotypes may shift when transmitted to a new host.

Transmissible spongiform encephalopathies (TSEs) are infectious diseases that affect humans and several livestock species, causing fatal neurodegeneration. TSEs are linked to the conversion of cellular prion protein (PrP^C^) to the aberrant form associated with the disease (PrP^SC^). Sheep scrapie, the most widely known TSE (*1*), has been documented in Europe for >2 centuries and is thought to have spread to other countries worldwide throughout the 1900s (*2*). Classical scrapie is caused by a variety of prion strains that can be distinguished by their biological and biochemical features (*3*), although several so-called atypical scrapie strains that have remarkably different biochemical and transmission characteristics have been recently described (*4*,*5*). Other TSEs include bovine spongiform encephalopathy (BSE), which reached epidemic proportions in Europe at the end of the past century due to the use of animal feed containing BSE-contaminated feedstuffs (*6*). A human variant of BSE, called variant Creutzfeldt-Jacob disease (vCJD) (*7*), was discovered in 1994 and reported in 1996 as linked to the BSE epidemic in the United Kingdom and elsewhere.

No reports exist of naturally occurring TSEs in pigs. However, the experimental inoculation of pigs and transgenic mice overexpressing porcine PrP has indicated that swine are susceptible to BSE infection by the parenteral route, although with a considerable transmission barrier (*8*,*9*). The oral transmission of BSE in pigs has not been demonstrated to date.

The potential spread of BSE to animals in the human food chain such as sheep, goats, and pigs needs assessing because a risk for human infection by animals other than BSE-infected cattle cannot be excluded. Moreover, the use of pigs as graft donors could cause concern, given a recent report of vCJD in the recipient of a porcine dura mater graft (*10*).

The transmission barrier limits TSE infection between different species. Sheep can be experimentally infected with BSE that is not easily distinguished from some scrapie strains showing a 19-kDa atypical proteinase K–resistant PrP (PrP^res^) unglycosylated band (*11*–*13*). Susceptibility and resistance to TSE infection in sheep is determined by polymorphisms at PrP amino acid positions 136, 154, and 171; sheep have the VRQ and ARQ alleles that are most susceptible to scrapie infection (*14*). Although ARQ is considered to show the highest susceptibility to BSE infection (*15*), the ARR allele was until recently thought to confer full resistance to BSE and scrapie (*16**,**17*). However, the successful transmission of BSE prions to ARR/ARR sheep (*18*) and the detection of natural cases of classical scrapie in sheep with the ARR/ARR genotype (*19*) have shown that this resistance is penetrable. Moreover, the identification of previously unrecognized atypical scrapie strains in sheep with various genotypes, including ARR/ARR, further supports this statement (*20*,*21*).

Although only 1 case of BSE in a goat has been confirmed, several putative field cases of BSE infection affecting goats and sheep have been detected in Europe, and the infectious properties of the resulting TSEs are not well known (*22*,*23*). In addition, a rise in scrapie outbreaks among flocks in Europe has been described; it is possible that some cases of alleged sheep scrapie could be ovine BSE. In a previous report, we demonstrated that BSE experimentally passaged in homozygous ARQ sheep showed enhanced infectivity (compared with cattle BSE) as determined in transgenic mice expressing bovine PrP protein (*24*).

Previous experiments showed that transgenic mice expressing porcine PrP (PoPrP-Tg001) can be infected with cattle BSE, but that infection is limited by a strong barrier (*8*): only some BSE inocula were able to infect PoPrP-Tg001 mice in primary transmission experiments, and when transmission occurred only a reduced percentage of the inoculated mice were affected. In the present study, we used the PoPrP-Tg001 mouse model to compare the porcine PrP transmission barrier to BSE infection before and after passage in sheep. In parallel, we also analyzed the susceptibility of PoPrP-Tg001 mice to a broad panel of scrapie isolates from different ovine PrP genotypes and with different biochemical characteristics.

## Materials and Methods

### Transgenic Mice

The PoPrP-Tg001 mouse line was generated and characterized as previously described (*8*). These mice express porcine PrP protein under the control of the murine PrP promoter in a murine PrP0/0 background. The animals express ≈4× the level of porcine PrP in the brain compared with the levels expressed in pig brains.

### TSE Isolates

#### Cattle BSE

Three isolates of different origins were used: cattle-BSE1, a pool of material from 49 BSE-infected cattle brains (TSE/08/59) supplied by the Veterinary Laboratory Agency (New Haw, Addlestone, Surrey, UK); cattle-BSE2, material obtained from the brainstem of 1 cow naturally infected with BSE supplied by the same agency (RQ 225:PG1199/00); and cattle-BSE0, an isolate obtained from the brainstem of 1 cow naturally infected with BSE (case 139) supplied by Institut National de la Recherche Agronomique (INRA) (Nouzilly, France).

#### Sheep BSE0

Sheep BSE0 came from a pool of brainstems from 7 ARQ/ARQ sheep experimentally infected by intracerebral inoculation with the same cattle-BSE0 described above. That work was part of the project “BSE in sheep” QLRT-2001-01309 (INRA, Nouzilly, France).

#### Sheep Scrapie Isolates

Eight scrapie isolates of different origins and biochemical characteristics obtained from sheep with different PrP genotypes were also used in this study. These isolates were SC-UCD-99, obtained from the brainstem of an Irish ARQ/ARQ sheep naturally infected with scrapie (provided by the Veterinary Research Laboratory, Abbotstown, Ireland); SC-Langlade, obtained from the brainstem of an ARQ/ARQ sheep from France naturally infected with scrapie (provided by INRA, Toulouse, France); SC-N662-97, obtained from the brainstem of an ARQ/ARQ sheep from Spain naturally infected with scrapie; SC-JR01, obtained from the brainstem of an infected VRQ/VRQ sheep provided by J. Requena (Santiago de Compostela University, Santiago de Compostela, Spain); SC-PS13, obtained from the brainstem of an ARQ/ARQ sheep from France naturally infected with scrapie (provided by INRA, Toulouse); SC-PS48, obtained from the brainstem of a VRQ/VRQ sheep from France naturally infected with scrapie (provided by INRA, Toulouse); SC-PS83, obtained from the brainstem of a ARR/ARR sheep from France naturally infected with scrapie (provided by INRA, Toulouse [*19*]); and SC-PS152, obtained from the brainstem of a AfRQ/AfRQ sheep from France naturally infected with atypical (Nor98-like) scrapie (provided by INRA, Toulouse).

SC-UCD/99 adapted to BoPrP-Tg110 was obtained after 2 subpassages of the SC-UCD-99 isolate in BoPrP-Tg110 mice expressing bovine PrP (*24*). For subpassages, equivalent amounts of brain homogenates from all PoPrP-Tg001 mice collected from primary passage were pooled and used as inocula. Brainstem from healthy homozygous ARQ sheep was inoculated in PoPrP-Tg001 mice as a negative control.

All inocula were prepared in sterile 5% glucose as 10% homogenates. To minimize the risk for bacterial infection, we preheated inocula for 10 min at 70ºC before inoculation.

### Transmission Studies

Groups of 12–20 mice (6–7 weeks of age) were housed according to the guidelines of the Code for Methods and Welfare Considerations in Behavioural Research on Animals (Directive 86/609EC). Mice were inoculated in the right parietal lobe by using a disposable 25-gauge hypodermic needle. Twenty microliters of 10% brain homogenate, containing similar amounts of PrP^res^ (as estimated by Western blot), was delivered to each animal.

The neurologic status of the inoculated mice was assessed twice a week. The presence of 3 of 10 signs of neurologic dysfunction established diagnostic criteria (*25*) was needed to score a mouse positive for prion disease. The animals were killed for ethical reasons when progression of the disease was evident or when considered necessary due to old age (650 days), and their brains were harvested for subsequent biochemical and histologic analysis.

### PrP^res^ Assay

Frozen brain tissue samples from mice were homogenized in 5% glucose in distilled water by using grinding tubes (Bio-Rad Laboratories, Hercules, CA, USA) and adjusted to 10% (wt/vol) with TeSeE Precess 48 Ribolyser OGER (Bio-Rad) according to the manufacturer’s instructions. Brain samples were analyzed by using the TeSeE Western Blot 355 1169 kit (Bio-Rad) but with some adjustments for the different amount of sample used. To arrive at the volume proposed in the manufacturer’s recommendations, 100 μL of the 10% brain homogenates to be tested was supplemented with 100 μL of 10% brain homogenate from PrP null mice (*26*). Processed samples were loaded on Criterion 12% acrylamide gels (165.6001; Bio-Rad) and electrotransferred to immobilon membranes (IPVH 000 10; Millipore, Billerica, MA, USA). For the immunoblotting experiments, Sha31 (*27*) and 12B2 (*28*) monoclonal antibodies (MAbs) were used at concentrations of 1 μg/mL. Immunocomplexes were detected by horseradish peroxidase–conjugated anti-mouse immunoglobulin G (Amersham Pharmacia Biotech, Piscataway, NJ, USA). Immunoreactivity was visualized by chemiluminescence (Amersham Pharmacia Biotech).

### Lesion Profiles and Paraffin-embedded Tissue Blots

All procedures involving mouse brains were performed as previously described (29). Briefly, samples were fixed in neutral-buffered 10% formalin (4% formaldehyde) before paraffin embedding. Once deparaffinated, 2 μm–thick tissue sections were stained with hematoxylin and eosin. Lesion profiles were established according to the standard method described by Fraser and Dickinson (*30*). For paraffin-embedded tissue (PET) blots, the protocol described by Andréoletti et al. (*31*) was used.

## Results

### Biochemical Properties of TSE Isolates

**Figure 1:**
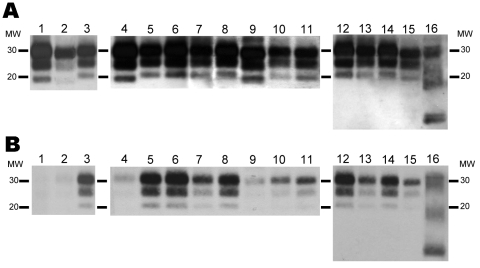
Electrophoretic profiles and antibody labeling of atypical proteinase K–resistant prion protein (PrP^res^) detected with monoclonal antibodies Sha31 (A) and 12B2 (B) in different isolates used for inoculating porcine PrP transgenic mice. Panels A and B were loaded with the same quantities of extracted PrP^res^ from each sample. MW, molecular mass in kilodaltons.

Samples of each TSE isolate were processed and analyzed by Western blotting. As shown in Figure 1, PrP^res^ in sheep BSE showed the characteristic unglycosylated band of 19 kDa (*32*); when compared with the original cattle BSE0 isolate, only slight differences could be observed in terms of the electrophoretic mobility of the PrP^res^, probably due to PrP amino acid sequence differences. ARQ/ARQ isolates SC-UCD/99, SC-Langlade, and SC-662/97 showed a PrP^res^ unglycosylated band of 21 kDa, and isolate SC-PS13 from the same sheep PrP genotype showed a smaller band of 20 kDa. The SC-JR01 and SC-PS48 isolates from a VRQ/VRQ sheep showed unglycosylated bands of 21 kDa and 19 kDa, respectively. An unglycosylated band of 21 kDa was detected in the ARR/ARR SC-PS83 isolate. Finally, the SC-PS152 isolate, whose genotype is widely associated with Nor98 cases (*33*,*34*), showed the characteristic band pattern of the atypical (Nor98-like) scrapies (*4*,*35*).

All scrapie isolates showing a 20–21-kDa unglycosylated band and the atypical SC-PS152 isolate were recognized by the Sha31 antibody (Figure 1, panel A) and the 12B2 antibody (Figure 1, panel B), which probe the WGQGG epitope (amino acids 93–97 of sheep PrP). However, SC-PS48 and sheep-BSE (showing a 19-kDa unglycosylated band) were poorly recognized by 12B2 MAb (Figure 1, panel B), suggesting that the 12B2 epitope is not protected against digestion with proteinase K in these isolates, as in cattle BSE.

### Susceptibility of PoPrP-Tg001 Mice to TSE Isolates

To evaluate the susceptibility of PoPrP-Tg001 mice to ARQ sheep BSE as opposed to the original cattle BSE, the BSE agent was inoculated in parallel before and after passage in ARQ/ARQ sheep in these mice. As shown in the Table, all PoPrP-Tg001 mice survived the cattle BSE0 infection and were culled without clinical signs at 650 days postinoculation (dpi), but when assessed for the presence of PrP^res^ in the brain, 3 (19%) were positive. In the second passage, all mice died at 197 ± 4 dpi. Similar results were obtained when 2 other BSE inocula (cattle BSE1 and cattle BSE2) were used (Table). In contrast, in PoPrP-Tg001 mice inoculated with BSE passaged in ARQ sheep (sheep BSE), an attack rate of 100% and survival time of 458 ± 11 dpi were observed, indicating that the PoPrP-Tg001 mice were fully susceptible to sheep BSE. Secondary subpassage in these mice led to a considerable reduction in the survival time (162 ± 4 dpi), which was maintained in subsequent subpassages. These results suggest the increased infectivity of BSE after passage in sheep in the PoPrP-Tg001 mouse model.

**Table:** Transmission of cattle BSE, sheep BSE, and sheep scrapie isolates in transgenic mice expressing porcine prion protein*

TSE isolate	Genotype	PrP^res^, kDa	First passage			Second passage			Third passage	
			Survival time, dpi	% (Attack rate)		Survival time, dpi	% (Attack rate)		Survival time, dpi	% (Attack rate)
Cattle-BSE1	–	19	>650	0 (0/12)†		269 ± 3	100 (10/10)		204 ± 12	100 (9/9)
Cattle-BSE2	–	19	498 ± 9	17 (2/12)†		198 ± 6	100 (15/15)		193 ± 17	100 (6/6)
Cattle-BSE0	–	19	>650	19 (3/16)†		197 ± 4	100 (12/12)		190 ± 10	100 (7/7)
Sheep-BSE_0_	ARQ/ARQ	19	458 ± 11	100 (15/15)		162 ± 4	100 (13/13)		166 ± 7	100 (7/7)
SC-662/97	ARQ/ARQ	21	>650	0 (0/10)		>650	0 (0/12)		ND	ND
SC-UCD/99	ARQ/ARQ	21	>650	0 (0/12)		>650	0 (0/9)		ND	ND
SC-Langlade	ARQ/ARQ	21	>650	0 (0/12)		>650	0 (0/12)		ND	ND
SC-PS13	ARQ/ARQ	20	>650	0 (0/12)		>650	0 (0/12)		ND	ND
SC-JR01	VRQ/VRQ	21	>650	0 (0/12)		>650	0 (0/12)		ND	ND
SC-PS83	ARR/ARR	21	>650	0 (0/12)		>650	0 (0/12)		ND	ND
SC-PS48	VRQ/VRQ	19	>650	0 (0/9)		>650	0 (0/10)		ND	ND
SC-PS152	AfRQ/AfRQ	≈7–14	300–600	16 (2/12)		162 ± 13	100 (9/9)		172 ± 16	100% (7/7)
SC-UCD/99 adapted to BoPrP-Tg110	–-		>650	0 (0/13)		>650	0 (0/10)		ND	ND
Healthy sheep brain	ARQ/ARQ		>650	0 (0/14)		>650	0 (0/9)		ND	ND

*BSE, bovine spongiform encephalopathy; TSE, transmissible spongiform encephalopathy; PrP^res^, atypical proteinase K–resistant prion protein; dpi, days postinoculation; ND, not determined. †No. animals positive for the aberrant form of PrP associated with disease / no. inoculated animals.

To evaluate the susceptibility of PoPrP-Tg001 mice to other sheep TSEs, we inoculated these mice with a panel of sheep scrapie isolates from different genotypes and with different strain properties. As shown in the Table, the atypical scrapie SC-PS152 isolate was the only one able to infect PoPrP-Tg001 mice at a low attack rate (16%) and survival time of 300 to 600 dpi. Secondary passage rendered a 100% attack rate and survival time of 162 ± 13 dpi. The other scrapie isolates included in the panel were not transmitted in the PoPrP-Tg001 mice either in primary or subsequent passages. We verified that the infectivity of the different scrapie isolate used in this work was sufficiently high for efficient transmission in transgenic mice expressing ovine PrP (data not shown).

PoPrP-Tg001 mice inoculated with healthy homozygous ARQ sheep brain material as controls were also euthanized after 600 dpi without showing clinical signs after first and second passages. None were positive for PrP^res^ in their brains.

### Biochemical Characterization of PrP^res^ in Inoculated PoPrP-Tg001 Mice

**Figure 2:**
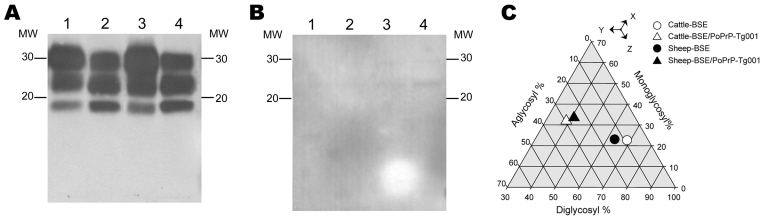
Brain atypical proteinase K–resistant prion protein (PrP^res^) of porcine PrP transgenic mice infected with cattle bovine spongiform encephalopathy (BSE) (lane 2) or sheep BSE agents (lane 4). Electrophoretic profiles and antibody labeling of PrP^res^ detected with monoclonal antibodies Sha31 (A) or 12B2 (B). Profiles produced by cattle (lane 1) and sheep BSE (lane 3) before passage in the porcine mouse model are shown for comparison. MW, molecular mass in kilodaltons. C) Triangular plot of the glycosyl fractions of PrP^res^ after proteinase K digestion and Western blotting using the Sha31 antibody. Data shown are the means of 5 or more measurements obtained from density scans in 2 or more Western blots. To interpret the plot, read the values for the diglycosyl, monoglycosyl, and aglycosyl fractions along the bottom, right and left axes of the triangle, respectively. For each point, the sum of the 3 values is 100.

PrP^res^ from BSE adapted to porcine PrP in PoPrP-Tg001 mice showed a different glycoprofile than the original inoculated BSE (Figure 2, panel C), although it preserved biochemical properties such as electrophoretic mobility (Figure 2, panel A) and lack of immunoreactivity to the 12B2 MAb (Figure 2, panel B). The 12B2 MAb is able to recognize porcine PrP^C^ as confirmed by Western blotting when samples not treated with proteinase K are used (data not shown).

In PoPrP-Tg001 mice, cattle BSE and sheep BSE agents produced identical PrP^res^ signatures and shared similar PrP^res^ biochemical properties (Figure 2, panel A). These features persisted after subsequent passages (data not shown).

**Figure 3:**
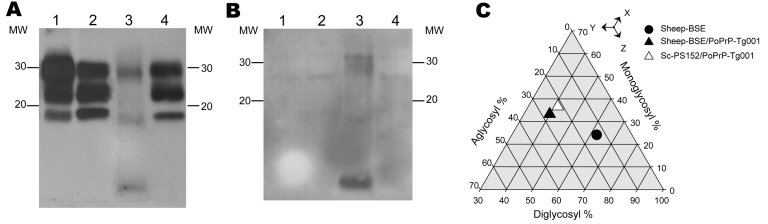
Brain atypical proteinase K–resistant prion protein (PrP^res^) of porcine PrP transgenic mice infected with an atypical scrapie (SC-PS152) agent (lane 4) versus sheep bovine spongiform encephalopathy (Sheep-BSE) agent (lane 2). Electrophoretic profiles and antibody labeling of PrP^res^ detected with monoclonal antibodies Sha31 (A) or 12B2 (B). Profiles produced by atypical scrapie (SCPS152) (lane 3) and sheep-BSE (lane 1) before passage in the porcine mouse model are shown for comparison. MW, molecular mass in kilodaltons. C) Triangular plot of the glycosyl fractions of PrP^res^ after proteinase K digestion and Western blotting using the Sha31 antibody. Data shown are the means of >5 measurements obtained from density scans in >2 Western blots. To interpret the plot, read the values for the diglycosyl, monoglycosyl, and aglycosyl fractions along the bottom, right and left axes of the triangle, respectively. For each point, the sum of the 3 values is 100.

In contrast, Western blot analysis of the porcine prion generated through the inoculation of atypical scrapie isolate (SC-PS152) showed a dramatic molecular shift after passage in the porcine PrP mouse model (Figure 3). A 3-band PrP^res^ pattern with an unglycosylated band of 19 kDa, which differed substantially from the molecular signature of atypical scrapie PrP^res^, was observed Moreover, this porcine prion was indistinguishable from the porcine prion generated by inoculation of sheep BSE in terms of PrP^res^ electrophoretic mobility (Figure 3, panel A), 12B2 MAb immunoreactivity (Figure 3, panel B), or its glycoprofile (Figure 3, panel C), characteristics that were maintained in subsequent passages in the same mouse model.

### Lesion Profile and PrPSc Deposition Pattern in Inoculated PoPrP-Tg001 Mice

**Figure 4:**
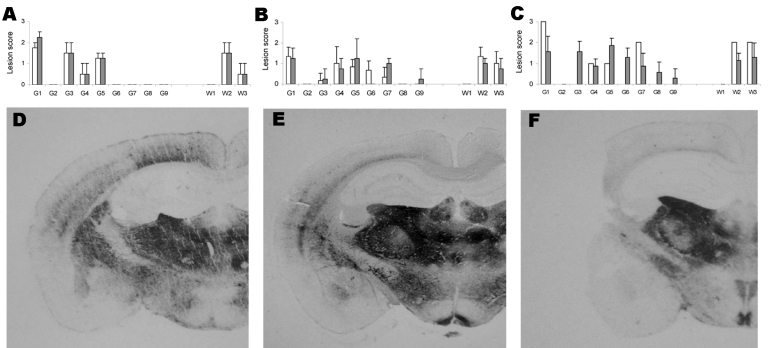
Lesion profiles and regional distributions of atypical proteinase K–resistant prion protein (PrP^res^) in the brain of porcine PrP transgenic mice infected, either in 1st passage (white column) or in 2nd passage (black column) with cattle bovine spongiform encephalopathy (BSE) (panels A and D), sheep BSE (panels B and E), or atypical scrapie (panels C and F) agents. A–C) Lesion scoring of 9 areas of gray matter (G) and white matter (W) in mice brains: dorsal medulla (G1), cerebellar cortex (G2), superior colliculus (G3), hypothalamus (G4), medial thalamus (G5), hippocampus (G6), septum (G7), medial cerebral cortex at the level of the thalamus (G8) and at the level of the septum (G9), cerebellum (W1), mesencephalic tegmentum (W2) and pyramidal tract (W3). Error bars indicate SE. D–F) Histoblots of representative coronal sections at the level of the hippocampus.

Brain material from PoPrP-Tg001 mice inoculated with the BSE-infected brains of either cattle or sheep showed consistent similarities in lesion profiles and PrP^Sc^ deposition patterns in PET blots, although some differences could be observed, mainly in areas G7 and G9. These features persisted after a second passage in PoPrP-Tg001 mice (Figure 4). PrP^Sc^ was also detected in the spleens of PoPrP-Tg001 mice inoculated with either cattle BSE or sheep BSE in first and second passages (data not shown). In mice inoculated with the different classical scrapie isolates, no typical brain lesions associated with prion infection were detected. We observed no differences between animals inoculated with classical scrapie isolates and those inoculated with brain tissue from healthy homozygous ARQ sheep or noninoculated PoPrP-Tg001 mice (data not shown).

Although some similarities were observed between the brains of PoPrP-Tg001 inoculated with SC-PS152 and the brains of those inoculated with cattle BSE and sheepBSE, some differences could be observed mainly in region G6 and G8 (Figure 4, panels A–C). Moreover, PrP^SC^ distributions in the PET blots showed some differences when compared with those observed in samples from both cattle and sheep BSE–inoculated mice (Figure 4, panels D–F). These differences mainly appeared in the cortex, the medial pretectal nucleus, the posterior commissure, the zona incerta and hypothalamic lateral area in which PrP^SC^ deposition was more intense in cattle and sheep BSE–inoculated mice than in the SC-PS152–inoculated animals.

## Discussion

In this study, transgenic mice expressing porcine PrP (*8*) were used to assess the transmission capacity of a wide range of TSE agents from sheep. Our results indicated that none of the classical scrapie isolates tested was transmitted to our porcine PrP mouse model after intracerebral inoculation (Table), suggesting a highly (if not completely) resistance to the classical scrapie strains tested independently of their origin and biochemical signature. The absence of successful transmission of the SC-PS48 isolates with an unglycosylated bands of 19 kDa-like BSE suggests a BSE-unrelated origin for these BSE-like scrapie strains.

The atypical isolate SC-PS152 was the only scrapie isolate able to infect the Po-PrP mouse model after intracerebral inoculation (Table), albeit with a low efficiency of infection in the first passage (attack rate 16%). These results suggest the potential ability of atypical scrapie prions to infect pigs, although with a strong transmission barrier. Given the increasing number of atypical scrapie cases found in Europe and in North America, the potential ability of atypical scrapie to adapt to the pig becoming more easily transmitted could raise concerns about the potential danger of feeding ruminant meat and bone meal to swine.

In our transmission experiments, an obviously shorter survival period (458 ± 11 dpi) and an increased attack rate (100%) were observed in PoPrP-Tg001 mice inoculated with sheep BSE (Table) compared with those inoculated with the original cattle BSE (>650 dpi, 19%). These last figures correlate well with those reported for other cattle BSE isolates (Table). Differences in survival times were maintained after subsequent passages in this mouse model (Table), suggesting that the increased infectivity of sheep BSE cannot be linked to a higher infectious titer in the initial inoculum but must be the outcome of a modification in the pathogenicity of the agent. We can also rule out that the primary amino acid sequence of the ovine PrP^SC^ leads to more efficient conversion of porcine PrP^C^ because scrapie isolates from sheep with the same ARQ-PrP genotype were not able to infect these mice (Table). Taken together, the increased infectivity of sheep BSE in the porcine PrP mouse model must be considered as increased pathogenicity of the agent attributable to its passage in sheep. These features support previous results indicating that the BSE agent modifies its biological properties after passage in sheep, with the result that its pathogenicity increases in transgenic mice expressing bovine PrP (*24*). An increased pathogenicity of ovine BSE was also reported in conventional RIII mice when compared with retrospective cattle BSE experiments (*36*). In other prion strains, passage through an intermediate species has also been noted to alter host susceptibility (*37*).

The enhanced infectivity of the BSE agent after its passage in ARQ sheep raises concern about its potential danger for other species, including humans. This question, as well as others related to the infectivity of the new porcine prion generated in this study, is currently being addressed in transmission experiments using transgenic mice expressing human PrP.

Upon passages in porcine PrP transgenic mice, the BSE agent retained most of its biochemical properties, except for its PrP^res^ glycoprofile in which some differences were appreciable. Our comparative analysis of cattle BSE and sheep BSE upon transmission in porcine PrP transgenic mice showed that both agents exhibit similar molecular (Figure 2) and neuropathologic properties (Figure 4). These features were preserved after subsequent passages. These results suggest that, despite their modified pathogenicity, the 2 porcine prions generated share the same biochemical and neuropathologic properties, regardless of whether the BSE agent used to inoculate the mice was obtained from ARQ sheep or cows. In agreement with these results, the increased infectivity of sheep BSE previously observed upon transmission in bovine PrP transgenic mice was not reflected in its molecular or neuropathologic properties (*24*).

The atypical scrapie (SC-PS152) agent appeared to undergo a strain phenotype shift upon transmission to porcine PrP transgenic mice. Surprisingly, this novel strain phenotype was similar to that of sheep BSE propagated in the same mice in terms of several features: 1) survival times observed after stabilization in PoPrP-Tg001 mice (second passages) were similar (Table); 2) PrP^res^ molecular profiles of the 2 agents in porcine PrP mice were indistinguishable (Figure 3); and 3) vacuolation profiles observed in second passages largely overlapped (Figure 4).

These findings could reflect the evolutionary potential of prion agents upon transmission to a foreign host able to promote strain shift and emergence of new properties (*38*,*39*). The converging molecular, neuropathologic, and biological properties of atypical scrapie and sheep BSE upon propagation in porcine transgenic mice could be the consequence of a restriction imposed by the porcine PrP^C^, which might only admit a few options as it changes its conformation to PrP^SC^.

Our results could also suggest a common origin for sheep BSE and atypical scrapie agents, which may exhibit different phenotypes depending on the host PrP^C^ or other host factors. Although this last explanation seems to be less likely, so far we cannot draw any definitive conclusion on this issue. Whichever the case, the ability of an atypical scrapie to infect other species and its potential capacity to undergo a strain phenotype shift in the new host prompts new concerns about the possible spread of this uncommon TSE in other species as a masked prion undistinguishable from other strains.
